# Characteristics, Classification, and Application of Stem Cells Derived from Human Teeth

**DOI:** 10.1155/2021/8886854

**Published:** 2021-05-31

**Authors:** Tong Lei, Xiaoshuang Zhang, Hongwu Du

**Affiliations:** ^1^Daxing Research Institute, University of Science and Technology Beijing, Beijing 100083, China; ^2^112 Lab, School of Chemistry and Biological Engineering, University of Science and Technology Beijing, Beijing 100083, China

## Abstract

Since mesenchymal stem cells derived from human teeth are characterized as having the properties of excellent proliferation, multilineage differentiation, and immune regulation. Dental stem cells exhibit fibroblast-like microscopic appearance and express mesenchymal markers, embryonic markers, and vascular markers but do not express hematopoietic markers. Dental stem cells are a mixed population with different sensitive markers, characteristics, and therapeutic effects. Single or combined surface markers are not only helpful for understanding the subpopulation of mixed stem cell populations according to cell function but also for improving the stable treatment effect of dental stem cells. Focusing on the discovery and characterization of stem cells isolated from human teeth over the past 20 years, this review outlines the effect of marker sorting on cell proliferation and differentiation ability and the assessment of the clinical application potential. Classified dental stem cells from markers and functional molecules can solve the problem of heterogeneity and ensure the efficacy of cell therapy strategies including dentistry, neurologic diseases, bone repair, and tissue engineering.

## 1. Introduction

Due to the advantages in immune privilege, ethical approval, and easy accession, mesenchymal stem cells are receiving increasing attention in medical research. Mesenchymal stem cells (MSCs) can be isolated from multiple human organs or tissues, such as umbilical cord blood, bone marrow, adipose tissue, and brain tissue. Dental stem cells are a kind of mesenchymal stem cells and can be obtained by specific methods, separating tissues around human teeth. So far, eight types of dental stem cells were successfully isolated, including dental pulp stem cells (DPSCs), stem cells from human exfoliated deciduous teeth (SHED), apical papilla stem cells (SCAP), periodontal ligament stem cells (PDLSCs), dental follicle stem cells (DFSCs), gingival mesenchymal stem cells (GMSCs), human tooth germ stem cells (TGPCs), and alveolar bone mesenchymal stem cells (ABMSCs) [1–3].

Obtaining the stability of the stem cell transplantation treatment effect has always been the core issue of clinical treatment [4–6]. The most fundamental problem is how to control the quality and efficacy of cell populations in regenerative medicine. This means that the safety of stem cell treatments can only be guaranteed by addressing cell population heterogeneity. However, even the subgroup of stem cells isolated from human teeth has significant differences in cell properties, such as proliferation and differentiation. Therefore, how to identify and characterize dental stem cells is an essential point in basic research, but the systematic discussion is lacking. This review attempted to combine the surface markers with the differentiation ability of eight types of dental stem cells and understand the feature of preclinical applications.

## 2. Dental Pulp Stem Cells

**Figure 1:**
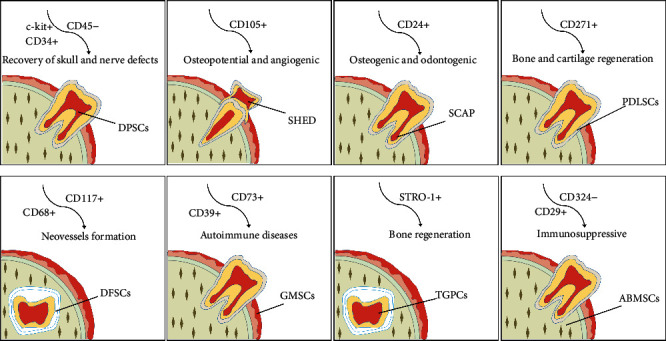
Schematic drawing demonstrating sources and markers of human dental stem cells with clinical application potential. DPSCs: dental pulp stem cells; SHED: stem cells from human exfoliated deciduous teeth; SCAP: stem cells from the apical papilla; PDLSCs: periodontal ligament stem cells; DFSCs: dental follicle stem cells; GMSCs: gingiva-derived mesenchymal stem cells; TGPCs: tooth germ progenitor cells; ABMSCs: alveolar bone-derived mesenchymal stem cells.

In 2000, inspired by the extraction of human bone marrow stromal cells (BMSCs), dental pulp stem cells (DPSCs) were first isolated from adult human dental pulp. DPSCs showed fibroblast-like cell morphology in Eagle's medium and a high colony formation activity as colony-forming unit-fibroblasts (CFU-F) (Figure 1) [7]. Surface marker analysis indicated that DPSCs were positive for CD29, CD44, CD73, CD90, CD105, CD117, CD146, CD271, CD166, and STRO-1, but negative for CD11b, CD14, CD19, CD34, CD45, CD79, CD106, and HLA-DR (Table 1) [8–12]. DPSCs were widely used in various clinical applications of regenerative medicine including dentin regeneration, treatment of retinal degeneration, spinal cord injuries, Parkinson's disease, Alzheimer's disease, cerebral ischemia, myocardial infarction, muscular dystrophy, and diabetes and immune diseases [13–16].

### 2.1. CD34, CD45, and c-Kit

CD34, CD45, and c-kit (CD117) are the markers of hematopoietic stem cells (HSCs). It has been found that CD34 is related to the short-term reproduction capacity and the formation of cell clones [17–19]. After planted in dental scaffolds, CD34 + DPSCs showed excellent biocompatibility, high proliferation viability, osteogenic differentiation, and high abundance of VEGF. This indicated that CD34 could be used as a marker to select DPSCs for subgroups that have the potential to become dental materials. CD45 is involved in proliferation and myogenic capacity [20, 21]. c-Kit is a receptor for stem cell factor (SCF) and c-kit+ HSCs can proliferate in response to hematopoietic factors [22, 23]. In enrichment analysis, these membrane molecules (CD34, CD45, and c-kit) are involved in cell adhesion, vasculature and bone development, cell proliferation, and stem cell differentiation. Populations of cells expressing CD34 and CD45, c-kit were identified as the first landmark in the evaluation of DPSCs. The c-kit+/CD34+/CD45-DPSCs that has high proliferation characteristics can not only differentiate into osteoblasts but also produce active autologous fibrous bone (LAB) tissue *in vitro*, which can form layered bone after being transplanted into immunodeficient rats [24].

### 2.2. STRO-1

The antibody of STRO-1 was found to bind to monocytes, and it can specifically identify antigens on perivascular cells in bone marrow and dental pulp tissues. STRO-1+ cell populations appeared to form CFU-F [25–27]. The expression of CD34 in DPSCs was controversial. STRO-1+/c-Kit/CD34- and STRO-1+/c-Kit+/CD34+ cells were similar in their differentiation capabilities. However, compared to STRO-1+/c-Kit+/CD34+ cells, STRO-1+/c-Kit+/CD34- cells proliferated faster and had less senescence and apoptosis. On the contrary, the STRO-1+/c-Kit+/CD34+ DPSCs expressed the typical neuronal markers, including CD271 and Nestin, and had a tendency toward neural differentiation [26]. STRO-1+/c-Kit+/CD34+ DPSCs are considered as a promising source of stem cells for the treatment of demyelinating disorders, which can improve the functional recovery of sciatic nerve defect in rats [28].

### 2.3. CD90 and CD271

CD90 is positive in mesenchymal progenitor cells but negative in skin fibroblasts. Based on the active screening of CD90, it was found that CD45-/CD90+ population showed a higher floating fat fraction and had a significant correlation with the frequencies of CFU-F [29–31]. CD271 expression is a screening criterion for hematopoietic progenitor cells. The CD34+/CD271+ population has high cloning activity [32]. CD90+/CD271+ DPSC subpopulations are able to form high-density cell populations. These subpopulations have the potential for long-term proliferation and multilineage differentiation. Hence, they can promote the formation of new bones and treat skull defects [33]. The cell surface hydrolase tissue nonspecific alkaline phosphatase (TNAP) (also known as MSCA-1), coexpressed with CD73 and CD90, is a potentially useful marker for DPSC selection, especially for mineralized tissue regenerative therapies [34]. CD271 is regarded as a marker of oral cells originating from the neural crest. This surface marker shows adipogenesis, cartilage formation, myogenic, and osteogenic potential of DPSCs [33, 35]. CD271-DPSCs display higher proliferation rates compared with CD271+ DPSCs [36].

## 3. Stem Cells from Human Exfoliated Deciduous Teeth

In 2003, stem cells from human exfoliated deciduous teeth (SHED) were isolated from normal detached incisors (Figure 1). Holistically, it is found that SHED express MSC-like antigens such as CD29, CD44, CD63, CD71, CD73, CD90, CD105, CD117, and CD146, CD166 as well as the embryonic stem cell markers OCT3/4, NANOG, SSEA-3, SSEA-4, TRA-1-60, and TRA-1-81, and progenitor cell markers CD13, CD31. Meanwhile, SHED do not express CD11b, CD14, CD18, CD19, CD31, CD34, CD45, CD49d, CD49e, CD106, CD133, CD184, CD197, and HLA-DR (Table 1) [37–40]. SHED can be induced into multilineage differentiation cell types including dopaminergic neurons, odontoblasts, osteoblasts, chondrocytes, adipocytes, liver cells, skin cells, and endothelial cells. SHED is a potential cell source in clinical applications such as dental regenerative, bone regeneration, intractable pediatric surgical diseases, liver failure, neural regeneration, and the revascularization for therapeutic applications [41–45].

### 3.1. CD105

CD105 (also known as Endoglin) is one of the positive markers of HSCs and skeletal-muscle-derived stem cells. CD105 can also promote the production of bone marrow and red blood cells, and regulate hematopoietic development of specific lineages. Besides, CD105+ MSCs have the adipogenesis and osteogenesis potential [46, 47]. In addition, CD105 is closely related to hereditary hemorrhagic telangiectasia and may be involved in preeclampsia, type 2 diabetes, and several types of cancer [48–51]. CD105 was validated in its ability to allow select and enrich for SHED in many studies, and CD105+ subpopulations in SHED have higher osteogenic potential *in vitro*. In addition, hsa-mir-1287 regulates CD105 expression and controls osteopotential in SHED by fine-tuning hsa-mir-1287 levels [52].

### 3.2. CD146

CD146, also known as MUC18, is a cell adhesion molecule. CD146+ MSCs tend to form bones, and the bone-forming ability will be downregulated in hypoxia [53, 54]. CD146 is often used as a marker to diagnose diseases such as systemic sclerosis, colorectal cancer, and breast cancer and as a target for immunotherapeutic treatment against osteosarcoma [55–57]. To obtain CD146+ and CD146- subpopulations of SHED, the magnetically activated cell sorting (MACS) was applied. The CD146 + SHED favors the differentiation of osteoblasts and glial and the negative subpopulation prefers adipogenic and neuronal differentiation [58]. Priming with FGF-2 is an effective way to increase the proportion of CD146+ cells in SHED [43].

## 4. Stem Cells from the Apical Papilla

Stem cells from the apical papilla (SCAP) were extracted from human immature permanent teeth in 2006 (Figure 1). SCAP is positive for CD24, CD29, CD34, CD45, CD73, CD90, CD105, CD106, CD146, CD166, and STRO-1 and negative for CD18, CD34, CD44, CD49f, CD81, CD117, and CD150 (Table 1) [59, 60]. SCAP shows immunosuppressive properties, which expand the application of SCAP in regeneration medicine, including immunotherapy and the reparation of multiple tissues (such as teeth, bone, nerve, and vascular tissue) [61].

### 4.1. CD24

CD24 is proposed as a specific surface marker, which can distinguish SCAP from DPSCs and BMSCs [60]. CD24 is strongly expressed in SCAP regardless of whether the tissue is inflamed or not, and one of the markers for multilineage stem cells in screening stable proliferation and differentiation populations [62, 63]. Moreover, CD24 shows a high abundance in the early stages of root development. It was found that high-CD24 expressing SCAP showed poor renewal ability but showed stronger osteogenic differentiation ability than low-CD24 expressing SCAP [64].

### 4.2. STRO-1 and CD146

One of the evidences for the presence of stem cells in the apical papilla was the positive expression of STRO-1 [65]. The positive expression of CD146 in SCAP is still controversial. The CD146+/STRO-1+ SCAP cell population was observed morphologically bigger with greater granularity in flow cytometry. Filtering by multiple marker combinations, the STRO-1+/CD146+ subpopulation was found to have a better potential for embryonic, mesenchymal, and odontogenic differentiation compared with the STRO-1-/CD146+ subpopulation [66, 67].

## 5. Periodontal Ligament Stem Cells

A cell population was collected by dissolving the periodontal ligament from a third molar of a 16-19-year-old human tooth to obtain a dispersed cell suspension, which could express MSC markers and differentiate into dentin cells, adipocytes, and collagen cells under special support nourishment. After transplantation, the cells were able to help rodents repair periodontal tissue, so the population was named as periodontal ligament stem cells (PDLSCs) (Figure 1). PDLSCs exhibited high expression of CD9, CD10, CD13, CD29, CD34, CD38, CD44, CD49d, CD59, CD73, CD90, CD105, CD106, CD146, CD166, and STRO-1, and lack of CD14, CD117, CD133, CD144, CD309, and HLA-DR (Table 1) [68–73]. PDLSCs can be physically stimulated to regulate proliferation and differentiation by activating different signaling pathways including MAPK, TGF/Smad, and Wnt/catenin pathways. This characteristic, PDLSCs fate to physical force *in vitro* and orthodontic force *in vivo*, determines that PDLSCs are a natural periodontal regeneration material in orthodontic tooth movement [74, 75].

### 5.1. STRO-1 and CD146

The STRO-1+/CD146+ PDLSCs population appears to be a unique cell population in humans while there are no such cells in mice [76]. Immunochemical staining is applied for STRO-1 and CD146 expression in PDLSCs. STRO-1+ PDLSCs can form colonies, which indirectly demonstrate that STRO-1 is an early progenitor cell marker for PDLSCs. Determined by flow cytometry, STRO-1+/CD146+ cells account for 2.6% of the population, and STRO-1-/CD146- cells account for more than 63% of PDLSCs. STRO-1+/CD146+ PDLSCs can form mineralized nodules, lipid vacuoles, and cartilage macromolecules after being induced under certain condition, demonstrating potent multilineage differentiation potential [69]. The outstanding ability to differentiate into the cartilage of STRO-1+/CD146+ PDLSCs making this population is a promising alternative material for cartilage regeneration.

### 5.2. CD271

CD271 plays an important role in tooth development and epithelial-mesenchymal transition (EMT). It is used as a universal receptor for neurotrophins to stimulate neuronal cell survival and differentiation [77, 78]. CD271+ PDLSCs demonstrate strong mineralization capabilities with the high expression of osteogenic markers such as DLX5, RUNX2, and BGLAP. CD271+ PDLSCs can be used as a promising bone regeneration material due to its excellent osteogenic differentiation ability. CD271 does not coexpress with CD90 and CD146, so it is used as a sole marker for sorting in PDLSCs [79].

## 6. Dental Follicle Stem Cells

Dental follicle stem cells (DFSCs) are separated from the tissue of the human dental follicle. Genes of FGFR1-IIIC, IGF-2, collagen 1, OCN, BS, and IGF-2 are more strongly expressed by the human DFSCs than in MSCs (Figure 1) [80]. Characterization of DFSCs reveals high expression of CD9, CD10, CD13, CD29, CD44, CD49d, CD56, CD59, CD90, CD105, CD106, CD166, and STRO-1 and poor expression of CD3, CD31, CD33, CD34, CD45, CD133, and CD271 (Table 1) [80–83]. DFSCs demonstrate the ability to differentiate multiple-lineages, such as osteoblastic/cementoblastic and neural lineages [84, 85].

### 6.1. CD68 and CD117

CD68 and CD117 are markers expressed by endothelial cells, and CD68 is a human macrophage marker [86]. CD117+ DFSCs may be involved in the signal transmission between cells during tooth germ development [87]. The expression of CD68 in DFSCs does not mean the presence of inflammatory cells. On the contrary, CD68+ DFSCs maintain a local hematopoietic stem niche. CD117+/CD68+ DFSCs can be used to identify endothelial progenitor cells and is related to a myeloid and progenitor phenotype in the human dental follicle. Meanwhile, the subsets are able to form neovessels and play a hematopoietic role [88].

## 7. Gingiva-Derived Mesenchymal Stem Cells

Gingiva-derived mesenchymal stem cells (GMSCs) are MSCs that reside in human gingival tissues (Figure 1). GMSCs express markers including CD13, CD44, CD73, CD90, CD105, CD146, CD166, CD271, and STRO-1, and not express CD14, CD19, CD34, CD38, CD45, and CD54 (Table 1) [89–94]. GMSCs maintain normal karyotype and telomerase activity in long-term *in vitro*, showing good clinical safety [95].

### 7.1. CD39 and CD73

CD39 and CD73 are coexpressed on GMSCs and regulate T cells, which play a catalytic role to promote immune regulation and reduce inflammation [96, 97]. CD39 and CD73 are involved in the formation of osteoclasts in GMSCs and significantly attenuate the severity of arthritis *in vivo* and *in vitro* [98]. Simultaneously, in the experiment of cotransplantation of GMSCs and PBMC in the NOD/SCID mouse, it was found that GMSCs suppressed the immune response through the CD39/CD73 pathway to relieve graft-versus-host disease (GVHD). It shows that CD39/CD73 can be used as a marker to evaluate the therapeutic effect of GMSCs on autoimmune diseases [99, 100].

## 8. Tooth Germ Progenitor Cells

A new type of dental stem cell was isolated and named as the tooth germ progenitor cells (TGPCs) in 2008 (Figure 1). RT-PCR and flow cytometry analysis tentatively suggest that TGPCs are positive expression of CD29, CD44, CD73, CD90, CD105, and CD166, and extremely negative expression of STRO-1, CD14, CD34, CD45, CD133, and HLA-DR (Table 1) [101–103]. TGPCs are able to differentiate into muscle, cartilage, fat, nerve, bone, and tooth, and it is an alternative material in regenerative medicine [104].

### 8.1. STRO-1

STRO-1 is positively expressed in TGPCs, and the subpopulation shows remarkable osteogenic differentiation ability. SRTO-1+ TGPCs exhibit strong mineralization with high expression of the osteogenic gene including OCT4, SOX2, MYC, and NANOG. STRO-1 can be used to evaluate the feasibility of TGPCs application in bone regeneration material [105, 106].

## 9. Alveolar Bone-Derived Mesenchymal Stem Cells

In 2005, alveolar bone mesenchymal stem cells (ABMSCs) were obtained from surgery on 6 to 44-year-old patients (Figure 1). ABMSCs are found to have sufficiently high proliferative capacity and myogenic potential. ABMSCs are positive expressions of CD29, CD44, CD73, CD90, CD105, CD146, and STRO-1, and negative for CD11b, CD19, CD31, CD34, CD45, and CD324 (Table 1) [107–110]. Protein arrays were used to identify the contents of ABMSCs and found that ABMSCs expressed IL-6 and MCP-1. ABMSCs are able to inhibit the activation and proliferation of monocytes and T cells after cocultured with different types of immune cells including THP-1 monocytes, macrophages, and peripheral blood mononuclear cell (PBMCs). Therefore, ABMSCs are a viable cell source for treating inflammation [111].

## 10. Regenerative Application of Dental Stem Cells

Dental stem cells are a potential enhancer of regenerative medicine in the future. Oral tissue repair and regeneration are the most widely clinical applications of stem cells in clinical trials (https://clinicaltrials.gov/ct2/home), including dental pulp regeneration, periodontal disease, peripical, periodontitis, knee osteoarthritis, dental plaque, alveolar bone atrophy, root canal therapy, and endodontic disease [112–114]. The use of biomaterials can enhance the efficiency of osteogenic differentiation *in vitro* and *in vivo*, including hydroxyapatite-tricalcium phosphate (HA-TCP) and demineralized dentin matrix (DDM) [115]. Dental stem cells can be induced into osteogenic, adipogenic, and neural differentiation in a specific medium [116]. Dental stem cells with pretreatment are an effective material such as in neurodegenerative diseases. Neuron-like stem cell transplantation can exert a stable and significant effect in brain diseases, which involves the activation of Wnt/*β*-catenin signaling, Rho kinase, and AKT/GSK3*β* signaling pathway [117–119].

The safety of stem cell transplantation is the primary requirement for cell transplantation [120]. The tumorigenicity of cell transplantation must be checked in clinical trials. Although the safety of dental stem cells has not been questioned, every clinical application should ensure safety under long-term monitoring and large-scale samples [121]. Few clinical records of dental stem cells have been retrieved, so the safety of human trials needs to be constantly monitored [122].

Due to the uncertainty of the timeliness of cell transplantation in vivo, the injection method is one of the factors that affect the therapeutic effect, including systemic injection and local injection [123]. Injection in the lesion area is one of the common ways to improve the effectiveness of cell transplantation, but it is not suitable for systemic diseases and chronic diseases [124, 125].

Why can cells improve symptoms and cure diseases? This is the most urgent problem to be solved in future research. The explanation of the mechanism of action can not only find safe and effective disease treatment drugs but also help to understand the pathogenesis of the disease [126]. Complex networks exist in the mechanism of various diseases, but the selection of key nodes needs to be supported by huge work.

## 11. Discussion and Conclusions

Since the beginning of the 21st century, stem cells isolated from teeth have been continuously discovered and reported. Dental stem cells exhibit outstanding neural differentiation and immune characteristics due to the neural crests origin. Ethical concerns regarding dental stem cells are not an issue, and the resolution of heterogeneity problems can help improve their performance and expand their applications [127]. One significant defect of stem cells isolated *in vitro* is the heterogeneity of the cell population. One of the main manifestations is the instability of the source of cellular materials, including the characteristics of surface markers and the ability to differentiate. On the other hand, the unstable treatment effect of stem cell transplantation is the biggest limitation of clinical application [128].

To supplement the practical requirement of allogeneic stem cell transplantation, the screening and identification of markers in dental stem cells have been widely carried out from different donors [129]. The analysis of stem cell contents and the composition of membrane molecules is one way to solve this problem. Proteomics has been applied to the characterization of stem cells, including two-dimensional electrophoresis, iTRAQ or TMT-labeled quantitative proteomics, and nanomass spectrometry [129–131]. However, studies have rarely analyzed the essential differences in one individual by omics analyses. Subsequent studies should concentrate on the differential expression of cells from the same donor [132].

Stem cell subpopulation studies will enable the control of stem cell heterogeneity by flow cytometry or sorting via magnetic beads. Membrane surface markers cannot represent functional cell populations. Stem cells capable of treating diseases should be characterized by functional molecules, which may be involved in metabolic physiological processes and signaling pathways to participate in pathogenesis and development [73, 105]. Surface molecules are used as markers to sort stem cell subsets. The proliferation and differentiation potential of subsets can be analyzed separately. Therefore, a sole marker or a combination of multiple markers separates a subset of cells with different characteristics [47, 50, 52, 54, 57, 67].

Stem cell-derived extracellular vesicles (EVs) are favored as an alternative to cell transplantation [133, 134]. EVs, including RNA, protein, and lipid metabolites, are able to circulate in the blood and cross the blood-brain barrier [135, 136]. EVs are considered to be functional vesicles and participate in disease regulation and are used as drug carriers [137, 138]. EVs secreted from DPSCs promote the formation of blood vessels and release of VEGF *in vitro* [139, 140]. It is believed that EVs can become potential clinical materials.

In conclusion, dental stem cells as a dental material have become a promising tool for solving oral clinical problems. The heterogeneity of dental stem cell populations severely hampers the process of elucidating their mechanisms of action. A sole marker or a combination of multiple markers is used to identify stem cells derived from teeth, which is an effective method to solve the problem of heterogeneity. In summary, the marker of stem cells is the first stage to ensure the effectiveness of cell therapy. Sole marker and associated markers of stem cells play an essential role in cell characterization in clinical applications. The resolution of heterogeneity problems can help improve performance and expand applications of dental stem cells.

## Figures and Tables

**Table 1:** Surface markers of human dental stem cells.

Marker	DPSCs	SHED	SCAP	PDLSCs	DFSCs	GMSCs	TGSCs	ABMSCs
CD3					—			
CD9				+	+			
CD10				+	+			
CD11b	—	—						—
CD13		+		+	+	+		
CD14	—			—		—	—	
CD18		—	—					
CD19	—	—				—		—
CD24			+					
CD29	+	+	+	+	+		+	+
CD31		+		+	—			—
CD33					—			
CD34	—	—	+	+	—	—	—	
CD38				+		—		—
CD39						+		
CD44	+	+	+	+	+	+	+	+
CD45	—	+	+	—	—	—	—	—
CD49d		—		+	+			
CD49f		—	+					
CD54						—		
CD56					+			
CD59				+	+			
CD63		+						
CD68					+			
CD71		+						
CD73	+		+	+		+	+	+
CD79	—							
CD81			+					
CD90	+	+	+	+	+	+	+	+
CD105	+	+	+	+	+	+	+	+
CD106		—	+	+	+			
CD117	+	+	+	—	+			
CD133		—	—	—	—		—	
CD140*α*				+				
CD140b				+				
CD144				—				
CD146	+	+	+	+				
CD150			—					
CD166		+	+	+	+	+	+	
CD184		—						
CD197		—						
CD271	+		+	+	—	+		
CD309				—				
CD324								—
STOR-1	+	+	+	+	+	+	+	+
HLA-DR	—	—		—			—	

## Abbreviations

MSCs: Mesenchymal stem cells
DPSCs: Dental pulp stem cells
SHED: Stem cells from human exfoliated deciduous teeth
SCAP: Stem cells from the apical papilla
PDLSCs: Periodontal ligament stem cells
DFSCs: Dental follicle stem cells
GMSCs: Gingiva-derived mesenchymal stem cells
TGPCs: Tooth germ progenitor cells
ABMSCs: Alveolar bone-derived mesenchymal stem cells
BMSCs: Human bone marrow stromal cells
HSCs: Hematopoietic stem cells
VEGF: Vascular endothelial growth factor
HLA-DR: Human leukocyte antigen-DR
iTRAQ: Isobaric tags for relative and absolute quantitation
GVHD: Graft-versus-host disease
CFU-F: Colony-forming unit-fibroblasts.
